# T cells loaded with magnetic nanoparticles are retained in peripheral lymph nodes by the application of a magnetic field

**DOI:** 10.1186/s12951-019-0440-z

**Published:** 2019-01-22

**Authors:** Laura Sanz-Ortega, José M. Rojas, Ana Marcos, Yadileiny Portilla, Jens V. Stein, Domingo F. Barber

**Affiliations:** 1 Department of Immunology and Oncology, and NanoBiomedicine Initiative, Centro Nacional de Biotecnología (CNB)-CSIC, Darwin 3, Cantoblanco, 28049 Madrid Spain; 2 Theodor Kocher Institute, University of Bern, 3012 Bern, Switzerland; 3 Present Address: Animal Health Research Centre (CISA)-INIA, Instituto Nacional de Investigación y Tecnología Agraria y Alimentaria, Valdeolmos, 28130 Madrid Spain; 4 Present Address: Section of Medicine, Department of Oncology, Microbiology and Immunology, University of Fribourg, 1700 Fribourg, Switzerland

**Keywords:** Cell-based therapy, T cell, Magnetic nanoparticle, Magnetic retention, Lymph node

## Abstract

**Background:**

T lymphocytes are highly dynamic elements of the immune system with a tightly regulated migration. T cell-based transfer therapies are promising therapeutic approaches which in vivo efficacy is often limited by the small proportion of administered cells that reaches the region of interest. Manipulating T cell localisation to improve specific targeting will increase the effectiveness of these therapies. Nanotechnology has been successfully used for localized release of drugs and biomolecules. In particular, magnetic nanoparticles (MNPs) loaded with biomolecules can be specifically targeted to a location by an external magnetic field (EMF). The present work studies whether MNP-loaded T cells could be targeted and retained in vitro and in vivo at a site of interest with an EMF.

**Results:**

T cells were unable to internalize the different MNPs used in this study, which remained in close association with the cell membrane. T cells loaded with an appropriate MNP concentration were attracted to an EMF and retained in an in vitro capillary flow-system. MNP-loaded T cells were also magnetically retained in the lymph nodes after adoptive transfer in in vivo models. This enhanced in vivo retention was in part due to the EMF application and to a reduced circulating cell speed within the organ. This combined use of MNPs and EMFs did not alter T cell viability or function.

**Conclusions:**

These studies reveal a promising approach to favour cell retention that could be implemented to improve cell-based therapy.
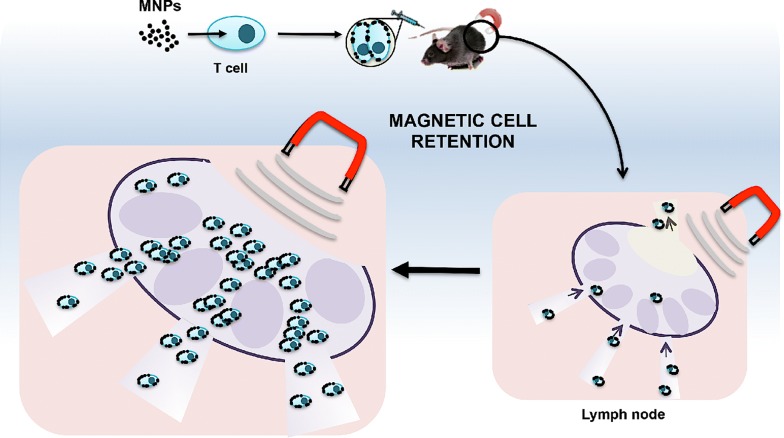

**Electronic supplementary material:**

The online version of this article (10.1186/s12951-019-0440-z) contains supplementary material, which is available to authorized users.

## Background

Immunotherapy has re-emerged as a promising therapeutic tool in recent years [1]. The idea of specifically modulating the immune response represents an attractive approach to restore or enhance the immune system’s ability to fight cancer or control autoimmune diseases. In particular, immune cell-based therapies, which are based on the use of the patient’s own cells after in vitro expansion and/or modification, are currently one of the most appealing strategies in this field [2, 3]. This approach can be applied to treat either cancer [4, 5] or autoimmunity [6–8]. The clinical response rates that these strategies elicit are nonetheless strongly correlated to the number of transferred cells that reach the desired region. As such, one of the main limitations of cell-based therapies is the dispersion of the in vivo-administered cells which results in only a small proportion of cells reaching the site of interest [9]. There is therefore a clear need to develop new strategies that promote specific cell infiltration, accumulation and survival in specific tissues so that they can exert their function effectively.

Nanotechnological approaches can offer a solution, as they can increase treatment effectiveness by concentrating therapeutic molecules in the required region. Nanoparticle-based drug delivery systems can access difficult-to-reach sites because of their small size. They can be directed by active or passive strategies based on the nanomaterial physical and chemical properties and/or through addition of targeting moieties in the nanoparticle coating [10]. One promising active approach is based on superparamagnetic iron oxide nanoparticles, which can be localized precisely in the desired area by applying an external magnetic field (EMF) [11]. This approach could promote specific cell accumulation and thereby improve the efficacy of cell transfer therapies. Non-lymphoid cells loaded with these magnetic nanoparticles (MNPs) can be inoculated systemically and attracted to a target tissue in mice by the application of an EMF [12–17]. These studies focused mainly on enriching stem cells, mesenchymal cells, macrophages or dendritic cells to control tissue injury and immune disorders. Studies using in vivo delivery of magnetically guided lymphoid cells to treat cancer and autoimmunity are very limited [18] and this field remains vastly unexplored. The manipulation of highly motile effector lymphoid cells such as T or natural killer (NK) cells to target and accumulate them to a specific region such as lymph nodes (LNs) or solid tumours could be a promising approach to enhance their activity in the desired area.

In this work, we focus on T lymphocytes, an essential effector cell of the adaptive immune system, which continuous circulation is very dynamic compared to other immune cells such as dendritic cells or macrophages. During an immune response, T cells need to interact with other cells and detect antigens in different contexts and tissues [19]. T cell migration, which is tightly regulated by multiple factors, such as cellular expression of selectins and chemokine receptors and interaction with other cells in secondary lymphoid organs (SLOs), is critical for the development of effective T cell responses [20, 21]. T cells circulate through lymphoid and target organs where they can search for their cognate antigen either on the surface of antigen-presenting cells (APCs) in lymphoid organs or on target cells in peripheral organs. Initial T cell activation steps occur in SLOs such as the LNs, where the tissue organization allows murine primary T cells to encounter their cognate antigen on APCs coming from different tissues and become activated. Once activated, these antigen-experienced T cells scan peripheral tissues to find and eliminate their antigen [22, 23]. Manipulating T cells to target specific locations could therefore be an interesting strategy to promote for instance their accumulation in the SLOs during priming and activation to enhance the immune response, or to favour their retention in the target tissue once activated. MNP-loading of T cells could thus be used for these targeting purposes as long as T cell function and interaction with chemokines, adhesion molecules and other cell types is not impaired by the treatment.

In this study, we evaluated the possibility that MNPs could be used as a platform to magnetically guide T cells to a region of interest. We assess T cell migration functionality after MNP treatment as well as their in vitro and in vivo manipulation to a target site through the application of an EMF.

## Methods

### Iron oxide core synthesis

Iron oxide cores were prepared by following the Massart coprecipitation protocol [24]. Briefly, 445 ml of a mixture of FeCl_3_·6H_2_O (0.09 mol) and FeCl_2_·4H_2_O (0.054 mol) were added to 75 ml of NH_4_OH (25%). This addition was performed slowly and under vigorous stirring. The mixture was heated at 90 °C for 90 min to prepare nanoparticles of approximately 12 nm in diameter. After that, the sample was washed three times with distilled water by magnetic decantation. To oxidize magnetite to maghemite and activate surface for subsequent steps, the precipitate was treated with 300 ml of HNO_3_ (2 M) under stirring for 15 min. Then, nitric acid was removed by magnetic decantation, and 75 ml of Fe(NO_3_)_3_ (1 M) and 130 ml of water were added over the preparation. The mixture was heated up to boiling temperature and stirred for 30 min. The particles were then cooled to room temperature and, by magnetic decantation, the supernatant was substituted by 300 ml of HNO_3_ (2 M) and stirred for 15 min. Finally, they were washed three times with acetone and dispersed in distilled water. A rotary evaporator was used to remove any acetone waste and concentrate the sample.

### MNP surface coating

Iron oxide cores were coated with dimercaptosuccinic acid (DMSA), 3-aminopropyl-triethoxysilane (APS) or dextran (6 kDa) following procedures previously described [25]. Briefly, for DMSA coating (DMSA-MNPs), 14.7 mg (0.08 mmol) of DMSA were added to a suspension of 30 ml of the iron oxide cores (0.05 M) under stirring at pH 3. After this, the pH was increased to 11 and the sample was sonicated for 20 min. After coating, this suspension was dialyzed for 2 days and finally; the pH was adjusted to 7. For APS coating (APS-MNPs), 1.17 ml (0.005 mol) of APS was added very slowly to a mixture of 10 ml of the iron oxide cores (28 g Fe_2_O_3_ per l) and 10 ml of methanol under vigorous stirring for 12–16 h. Methanol was eliminated from the mixture using a rotary evaporator. The sample was then washed three times with a mixture of acetone/water (70/30) and redispersed in 10 ml of distilled water. The pH was decreased to 3 and the sample was sonicated for 1 h. Finally, the pH was adjusted to 7 and sonicated for 10 min. For Dextran coating (DEXT-MNPs), the iron oxide core dispersion [600 mg of Fe_2_O_3_ in 4.8 ml NaOH (0.8 M)] was added dropwise into a solution of 600 mg of dextran (6 kD) in 7.5 ml NaOH (0.5 M) under sonication. The mixture was sonicated for 6 h under refrigeration. After coating, this suspension was dialyzed for 3 days and finally; the pH was adjusted to 7.

### MNP characterization

For TEM analyses, a drop of a dilute MNP suspension was placed on a carbon-coated copper grid and the solvent allowed to dry at room temperature. Images were acquired with a JEOL JEM 1011 transmission electron microscope with Gatan ES1000Ww camera. MNP size, shape and distribution were determined using TEM images and ImageJ software. For hydrodynamic size and Z-potential determination, we analysed a suspension of MNPs in water or in 0.01 M KNO_3_ solution, respectively. Dynamic light scattering (DLS) for colloidal characterization was carried out using a Nano Sizer ZS (Malvern).

Coating presence on MNP surface was determined from FTIR spectra, recorded between 4000 and 250 cm^−1^ on a Bruker (USA) IFS 66V-S spectrometer and a Nicolet FT-IR 20SXC (Thermo Scientific, USA). Thermal analysis was performed to quantify the amount of coating molecules on the MNP surface. Simultaneous thermogravimetric (TG) and differential thermal analysis (DTA) were performed on a Seiko TG/DTA 320U thermobalance (Seiko Instruments, Japan). Samples were heated from room temperature to 900 °C at 10 °C/min under an airflow of 100 ml/min. Iron determination was carried out in an inductively coupled plasma-optical emission spectrometry (ICP-OES) OPTIME 2100DV [Perkin Elmer (USA)] apparatus after acid digestion. For the magnetic characterization, liquid samples were frozen and dried overnight in a LyoQuest freeze dryer (Telstar, Spain). The resulting solid sample was compacted into gelatine capsules for magnetic characterization. Hysteresis loops with a maximum field of 5 T were measured in a Vibrating Sample Magnetometer (MLVSM9, MagLab 9T, Oxford Instruments, UK). AC magnetic susceptibility measurements were performed in a Quantum Design (USA) MPMS-XL SQUID magnetometer with magnetic field amplitude of 0.41 mT and a frequency of 11 Hz in the temperature range between 2 and 300 K. Endotoxin levels in MNP preparations were measured using a commercial LAL chromogenic assay (Lonza) according to the manufacturer’s instructions.

### Cell culture

The human cell line Jurkat (ATCC: TIB-152) and the murine cell line RAW264.7 (ATCC: TIB-71) were cultured in DMEM with 10% FBS, 2 mM l-glutamine, 1 mM sodium pyruvate, 50 µM 2-mercaptoethanol and 100 U/ml penicillin/streptomycin (P/S) in standard culture conditions (37 °C, 5% CO_2_, 90% relative humidity). Murine primary T cells were purified from spleen and LN cell suspensions obtained from C57BL/6 mice (Harlan Laboratories) using the Mouse T cell Isolation kit (STEMCELL Technologies). After isolation, T cells were 90–95% CD3^+^ by flow cytometry analysis and cultured in RPMI with 10% FBS, 2 mM l-glutamine, 2-mercaptoethanol and 100 U/ml P/S in standard culture conditions (37 °C 5% CO_2_, 90% relative humidity).

### MNP treatment

Jurkat and murine primary T cells were incubated with MNPs (150 µg Fe/ml) in a reduced volume at high cell density (10^6^ cells in 100 µl corresponding medium) for 2 h in standard conditions.

### Cell viability, MNP uptake and staining assays

Cell viability was analysed by two methods. In the AlamarBlue assay (Invitrogen), cells were cultured in a 96-well plate with different MNP concentrations for 24 h and AlamarBlue was added to each well, incubated for 4 h and fluorescence was finally measured (530 nm excitation, 590 nm emission). Cell survival is expressed as the percentage of fluorescence of MNP-treated cells compared to untreated cells. For FITC-annexin V/propidium iodide staining, cells were processed using the Annexin V-PI apoptosis assay kit according to the manufacturer’s protocol (Life Technologies) and analyzed by flow cytometry on a FC500 flow cytometer.

To quantify MNP uptake, cells (10^7^ cells/ml) were incubated with MNPs (150 µg Fe/ml) for 2 h in standard conditions, washed with PBS and digested for 1 h at 90 °C sequentially with 1 ml HNO_3_ 63% and then, with 1 ml H_2_O_2_. Iron determination was performed by ICP-OES as in MNP characterization.

Different microscopy techniques were used to determine MNP subcellular location. For iron staining, cells were washed with PBS after incubation with MNPs, fixed in paraformaldehyde (PFA) 4% (15 min), permeabilized with TritonX-100 (5 min), stained with an equal volume of HCl 4% and potassium ferrocyanide trihydrate 4% (Prussian blue, 20–30 min) and counterstained with neutral red 0.5% (1 min). Samples were washed with distilled water, air-dried and mounted using mounting medium (7.7% gelatine, 54% glycerol). Images were acquired on an Olympus IX70 inverted bright field microscope with 63× or 100× oil-objectives. For dark-field confocal microscopy, LysoTracker Red DND-99 (Life Technologies) was added to the media during the incubation with MNPs. Alexa Fluor 647-wheat germ agglutinin (Life Technologies) was added during the last 15 min to the culture. Cells were then washed, fixed with PFA 4% (15 min), counterstained with DAPI and mounted in Fluoromont-G (SouthernBiotec). Images were acquired with a 0.13 μm step with a confocal multispectral Leica TCS SP5 system with a 63×/1.4 NA oil objective and 5× zoom. For dark-field acquisition of MNPs, the 488 nm laser light was used. ImageJ software was used for image analysis and orthogonal projections. For TEM microscopy, cells were fixed at RT in 2% glutaraldehyde, 1% tanic acid in 0.4 M HEPES at PH 7.2, washed and resuspended in HEPES buffer. Samples were processed and included by the Transmission Electronic Microscopy Service at the National Center for Biotechnology (CNB-CSIC, Madrid, Spain). Images were acquired at different magnifications with a JEOL JEM 1011 transmission electron microscope with Gatan ES1000Ww camera.

### Real-time metabolic analysis

Metabolic profiling of MNP-free and -loaded murine primary T cells was undertaken using a Seahorse XFp Extracellular Flux Analyser (Agilent Technologies) with the Agilent Seahorse XFp Cell Energy Phenotype Test Kit following the manufacturer’s protocol.

### Calcium imaging assays

An 8-well μ-Slide (Ibidi) was coated with poly-lysine (Sigma) for 1 h at RT, washed 3 times with PBS and allowed to dry at 37 °C. Cells (10^7^ cells/ml) were incubated with MNPs (150 µg Fe/ml) for 2 h in standard conditions. Untreated and MNP-treated cells were washed with PBS and calcium starved for 3 h. After this, cells (2.5 × 10^6^ cells/ml) were stained with Fluo-3 AM (3 µM, Invitrogen) for 30 min at 37 °C in rotation and washed with HBSS 1× (Gibco) 10% FBS without calcium. 2.5 × 10^5^ cells in 250 µl of HBSS 1× 10% FBS without calcium were seeded per well and allowed to attach to the bottom of the poly-lysine coated slides for 5 min. Movies were acquired every 645 ms with a confocal multispectral Leica TCS SP5 system with a 20× objective and 2× zoom. Cell images were first recorded without calcium or stimuli for 3 min and then, 50 µl HBSS 1× 10% FBS with 50 ng/ml phorbol 12-myristate 13-acetate (PMA) (Sigma), 5 µM ionomycin (Sigma) and 2 mM CaCl_2_ were added and movies immediately acquired for 10 min. In some experiments, an 8 × 6 mm neodymium–boron–iron (NdFeB) permanent magnet (Br: 1.45 T) was placed next to the well to study EMF effects. The appropriate unstained and/or unloaded controls were also performed. LAS X Life Science Software (Leica) was used to analyse fluorescence fluctuation in cells due to calcium fluxes changes.

### Mice

C57BL/6 mice purchased from Harlan and athymic nude mice purchased from Envigo were maintained in the CNB animal facility and handled according to the recommendations of the CNB-CSIC institutional Ethics Committee. C57BL/6-Tg(CAG-EGFP)131Osb/LeySopJ (“Ubi-GFP”) mice purchased from Jackson and C57BL/6 mice purchased from Janvier were maintained in the Theodor Kocher Institute animal facility and handled according to the recommendations of the Cantonal Ethics Committee. The procedures involving animal work at CNB-CSIC were approved by the Ethics Committee for Animal Experimentation at the CSIC and by the Division of Animal Protection of the Comunidad de Madrid in compliance with national and European Union legislation. The procedures involving animal work at the Theodor Kocher Institute, University of Bern were approved by the Cantonal Committee for Animal Experimentation and were conducted according to federal and cantonal guidelines.

### Flow cytometry

Cells (10^7^ cells/ml) were incubated with MNPs (150 µg Fe/ml) for 2 h in standard conditions, washed with PBS and stained with fluorochrome-conjugated Abs against cell surface markers. For Jurkat cells, the following anti-human antibodies were used: anti-CD62L (DREGC 56, Coulter), -CD11a (25.3.1, Immunotech), -CD45 (KC56 (T-200), Beckamn C.) and -CD44 (G44-26, Pharmingen). For murine primary T cells, the following anti-mouse antibodies from Biolegend were used: anti-CD44 (IM7), -CD62L (Mel-14), -CD11a (M17/4), -CCR7 (4B12), -CD4 (RMA-5) and -CD8 (53-6.7). CCR7 labelling was performed at 37 °C for 30 min with biotinylated primary antibody followed by secondary staining with PE-conjugated streptavidin. Data were acquired on a FC500 flow cytometer or in Attune Nxt flow cytometer and analysed with FlowJo software.

### Transwell migration assay

Jurkat or murine primary T cells (10^7^ cells/ml) were treated or left untreated with MNPs (150 µg Fe/ml) for 2 h in standard conditions and washed with PBS. MNP-treated and untreated cells were differentially labelled with PKH26 Red or PKH67 Green fluorescent cell linker kits (Sigma Aldrich). MNP-treated and untreated cells were alternatively stained in red or green in repeat experiments to exclude dye labelling effects. MNP-treated and untreated cells were mixed in a ratio 1:1 and seeded (5 × 10^5^ cells in 0.1 ml) in each insert (Corning, 5 µm pore insert for Jurkat cells and 3 µm for murine T cells) in RPMI+ 2% FBS. To create a chemotactic gradient the recombinant human CXCL12 (10 nM, Peprotech) or the recombinant murine CCL21 (25 nM, Peprotech) was added to the lower chamber in Jurkat or murine T cells, respectively. Cells were allowed to migrate for 16 h (Jurkat cells) or 2 h (murine T cells) after which lower chamber contents were collected, stained with fluorochrome-labeled Abs against CD8 (53-6.7) and CD4 (RMA-5) in the case of murine T cells and cells were counted by flow cytometry, using a FC500 flow cytometer, and analysed by FlowJo software. Cell migration was referred and normalized to an input well [without transwell (100% migration)]. For magnetic field-exposed transwell assays, an 8 × 6 mm NdFeB permanent magnet (Br: 1.45 T) was placed under the well, just below the pore insert.

### Flow chamber assays

In vitro magnetic retention assays under flow conditions were performed in a modified channel slide (µ-Slide I Luer, 0.4 mm height, ibidi). This modification allowed the two-magnet system, specially designed for this assay, to be placed on the chamber and produce a homogeneous strong magnetic gradient on both sides of the channel. Jurkat or murine primary T cells (10^7^ cells/ml) were incubated or left untreated with different doses of MNPs (50, 100 and 150 µg Fe/ml) for 2 h in standard conditions, washed with PBS and stained with calcein-AM (ThermoFisher) for 20 min at 37 °C in PBS with 0.5% FBS. Cells were then diluted in PBS with 0.5% FBS (2.5 × 10^5^ cells/ml) until use. Flow chamber set-up was mounted on an Olympus Inverted Microscope model IX71 connected to an Imaging Station cell^R^ and in standard culture conditions (37 °C 5% CO_2_, 90% relative humidity). PBS with 0.5% FBS was infused at high flow rate to fill the system. Cells were infused at 0.5 dyne/cm^2^ (≈ 100 μl/min). Images were recorded every 1 s for 4 min. After the first 60 s, the two-magnet system was placed into the flow chamber and its effect recorded for the remaining time. IMARIS Software (Bitplane) was used to generate the movies and for cell motility tracking. Displacement in Y-axes (magnetic-field direction) was analysed.

### Adoptive transfer and in vivo T cell homing

Jurkat or murine primary T cells from C57BL/6 mice, MNP-treated and untreated, were stained with CFSE (Invitrogen) and Dye eFluor 670 (Thermofisher) and cell mixtures (1:1, total 10^7^ cells/100 µl) were injected intravenously into nude or C57BL/6 mice, respectively. Mice were sacrificed after 90 min or 24 h for Jurkat cell transfer, or 1 h for murine T cell transfer. Spleen, peripheral (inguinal and axillary) (PLN) and mesenteric LNs (MLN) were removed to obtain a single cell suspension, and fluorophore-labeled cells counted by flow cytometry [FACSCalibur flow cytometer (BD)] and analysed by FlowJo Software.

For magnetic-targeted homing, an 8 × 6 mm NdFeB permanent magnet (Br: 1.45 T) was placed over one of the popliteal LNs for 30 and 90 min for Jurkat cells and 20 min for murine T cells. In this case, axillary, inguinal and popliteal LNs from both sides were collected.

### Selective plane illumination microscopy (SPIM)

A mixture of MNP-treated and untreated murine primary T cells (10^7^ cells/mouse, ratio 1:1), isolated from the LNs and spleen of Ubi-GFP mice, were inoculated intravenously into C57BL/6 mice and 20-min homing experiments with an 8 × 6 mm NdFeB permanent magnet (Br: 1.45 T) over one of the popliteal LNs was performed. Untreated cells were previously stained with CMTMR (5 µM, Molecular Probes) for 20 min at 37 °C to differentiate them from the treated ones (only GFP-labelled). High endothelial venules (HEV) networks were labelled by intravenous injection of Alexa Fluor 633-conjugated MECA79 mAb (Nanotools) 10 min prior to sacrifice and LN harvest. At 20 min after T cell transfer, popliteal LNs were collected and fixed in PFA 4% for 24 h before removing adjacent fat tissue under a stereomicroscope. Cleaned LNs were mounted in 2% ultrapure low-melting agarose and were optically cleared following the CUBIC protocol [26]. After this, LNs were scanned using a multispectral SPIM setup as described [27].

### Two-photon laser scanning microscopy (2PM) of popliteal LNs

To analyse the behaviour of MNP-treated T cells in the LNs, the right popliteal LN of recipient mice was surgically prepared as previously described [28]. One or two 8 × 6 mm NdFeB permanent magnet (1.45 T) were placed as close as possible to the popliteal LN as showed in Fig. 9. Once prepared, a mixture of CMTMR- (5 µM) or CMAC- (20 µM) MNP-treated and untreated cells (10^7^ cells/mouse, ratio 1:1) was intravenously injected and HEV networks were labelled by intravenous injection of Alexa Fluor 633-conjugated MECA79 mAb (Nanotools) just prior to recording. CMAC (CellTracker Blue) and CMTMR (CellTracker Orange) were both purchased from Molecular Probes. 2PM imaging was performed using a TrimScope 2PM system equipped with a fluorescence microscope (BX50WI; LaVision Biotec; Olympus) equipped with a 20× objective (NA 0.95; Olympus). (11–15)-slice z stacks with 4-µm spacing of 250 × 250–µm field of views (FOVs) were acquired every 20 s for 30 min. IMARIS Software was used to generate 4D movies and for cell motility tracking. The Chemotaxis and Migration tool from Ibidi was used to plot the cells’ paths and analyse their directionality.

## Results

### Synthesis and characterization of MNPs with different surface charges for T cell guidance

**Fig. 1:**
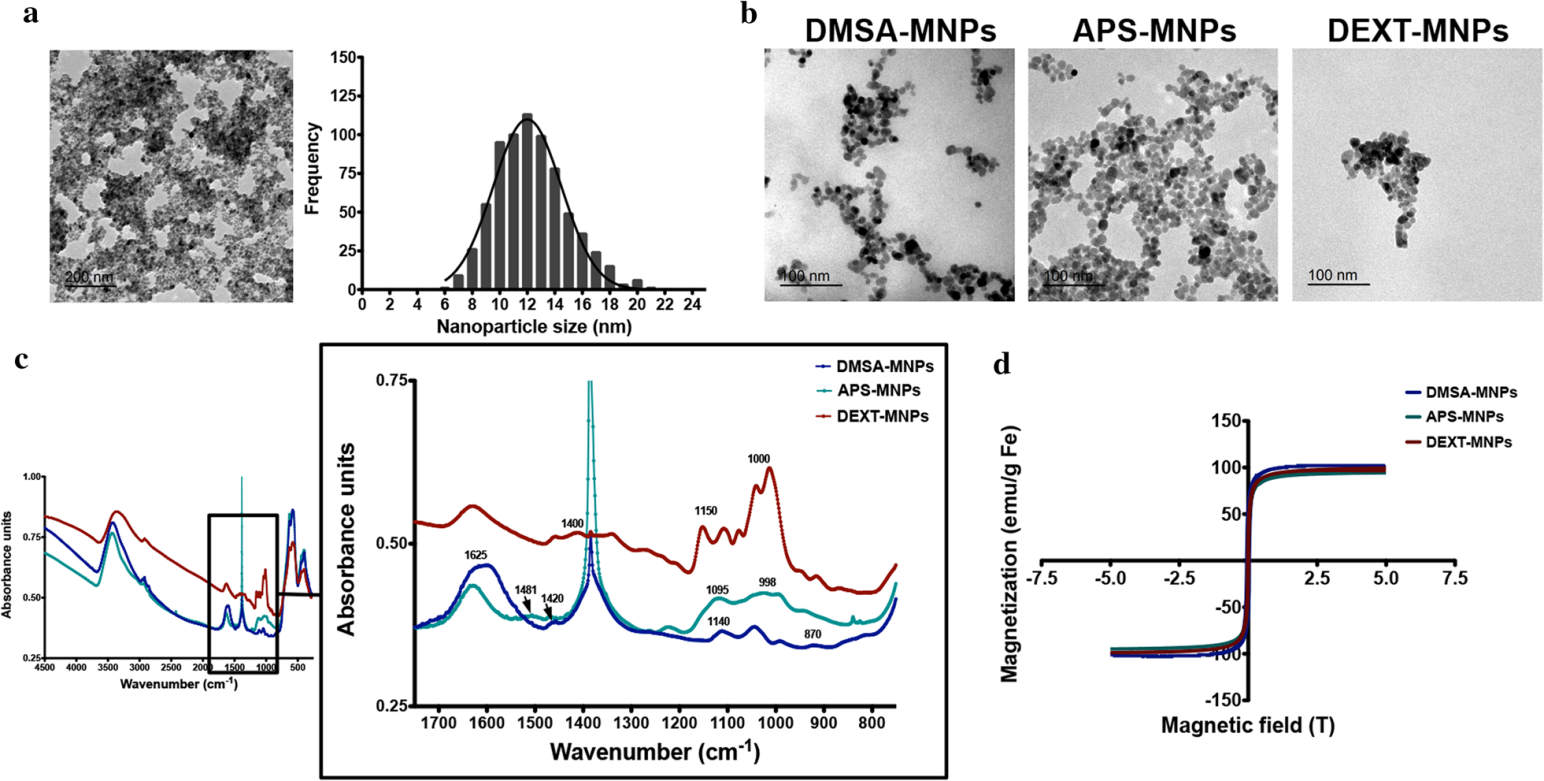
Physico-chemical characterization of MNPs. **a** TEM images of iron oxide cores prepared by co-precipitation synthesis and MNP size distribution and Gaussian fitting. **b** TEM images of DMSA-MNPs, APS-MNPs and DEXT-MNPs. **c** Fourier transformed infrared spectra for DMSA-MNPs, APS-MNPs and DEXT-MNPs. **d** Magnetization curve for DMSA-MNPs, APS-MNPs and DEXT-MNPs showing the superparamagnetic behaviour

MNPs with almost spherical morphology were obtained by the coprecipitation method and subsequent acid treatment (Massart’s procedure). Transmission electronic microscopy (TEM) imaging showed that these magnetic nanoparticle cores were on average 12.5 nm in diameter (Fig. 1a). The polydispersity degree was approximately 20%, indicating they are in the monodispersed range. MNPs were subsequently coated with dimercaptosuccinic acid (DMSA-MNPs), 3-aminopropyl-triethoxysilane (APS-MNPs) or dextran 6 kDa (DEXT-MNPs). This range of coatings produced MNPs with different surface charge and hydrodynamic size. The average particle size core of the coated MNPs and their aggregation status were verified by TEM (Fig. 1b). Fourier-transform infrared spectroscopy (FTIR) was used to evaluate the nature of each coating and its association to the MNP surface (Fig. 1c). Coated MNPs showed characteristic IR bands at frequencies between 1000 and 1400 cm^−1^ associated to C–O and C–C bonds due to polymer presence on MNP surface. Extra bands were detected, at 1625, 1400 and 1140 cm^−1^ for DMSA (corresponding to the carboxylic acid-iron bond and C=O bonds), at 1095 and 998 cm^−1^ for APS (corresponding to the formation of Si–O-Fe and Si–O-Si bonds) and in the range between 1000 and 1500 cm^−1^ and at 1400 cm^−1^ for dextran (due to C–O–C and C=O bonds and CH_2_ and C–O–H deformations) [25, 29]. IR bands at 500–600 cm^−1^ were present in all cases, due to the Fe–O bond (Fig. 1c). The band at 1381 cm^−1^ in APS-MNP spectra is due to the presence of nitric acid employed during MNP oxidation and pH adjustment [30]. Magnetization measurements confirmed MNP magnetic properties (Fig. 1d).

MNP hydrodynamic size values and surface charge were measured by dynamic light scattering (DLS). DMSA-MNPs, APS-MNPs and DEXT-MNPs hydrodynamic sizes were 83 nm, 82 nm and 120 nm respectively (Table 1). Coating quantification was performed by thermogravimetric (TG) analyses. The weight percentage of these coatings varied from 10% for DMSA-coated MNPs and APS-coated MNPs to approximately 38% for DEXT-coated MNPs (Table 1). Iron concentration in the MNP preparations was measured by inductively coupled plasma optical emission spectrometry (ICP-OES). Endotoxin levels were below 0.1 EU/ml for all MNP preparations (Table 1).

**Table 1:** Summary of the main characteristics of DMSA-MNPs, APS-MNPs and DEXT-MNPs

MNPs	Coating	Core diameter (nm)	Hydrodynamic diameter (nm)	Z-potential (mV)	Coating (%)	Endotoxin level (EU/ml)
DMSA-MNP	Dimercaptosuccinic acid	12.5	83	− 34	10	< 0.1
APS-MNP	3-Aminopropyl-triethoxysilane	12.5	82	+ 38	10	< 0.1
DEXT-MNP	Dextran 6 kDa	12.5	119	− 2	38	< 0.1

### MNPs are not toxic and are mainly detected on T cell surface

**Fig. 2:**
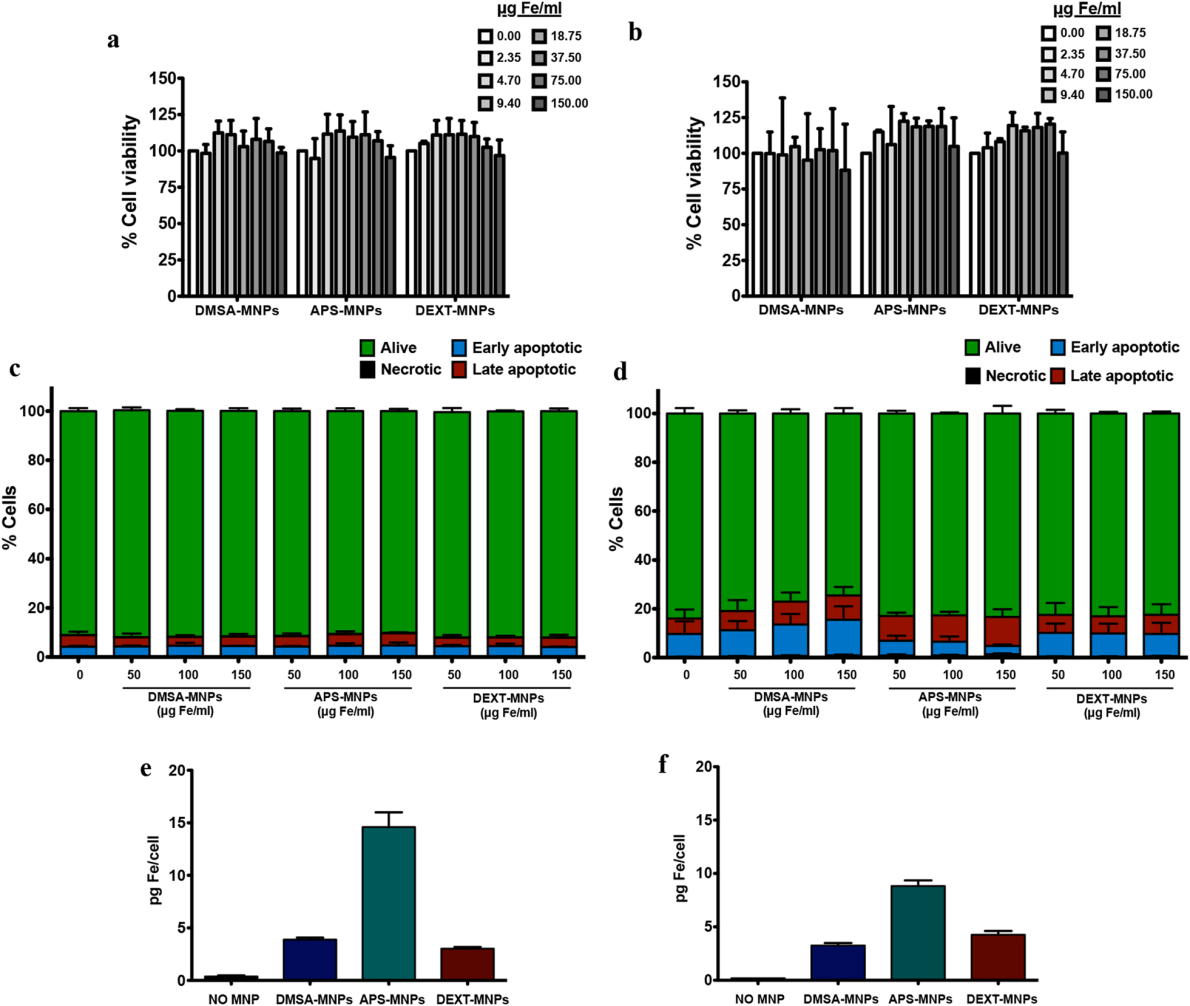
MNP toxicity and association to Jurkat and murine primary T cells. **a** Jurkat and **b** murine T cell viability after MNP treatment measured by AlamarBlue assays. Annexin-V/PI analysis by flow cytometry of **c** Jurkat and **d** murine T cells after MNP treatment. **e** Jurkat and **f** murine T cell iron association after MNP treatment. Data (mean ± SD) are representative of three independent experiments in all analyses

To assess MNP effects on T cell we chose two T cell models: the human T cell line Jurkat and murine primary T cells isolated from C57BL/6 mice. To examine nanoparticle toxicity, we first analysed cell survival after MNP incubation using different assays. MNP treatment did not affect Jurkat and murine T cells viability in AlamarBlue assays (Fig. 2a, b). Indeed, MNP-treated T cells often showed greater fluorescence readings in the AlamarBlue assays, indicating a slight increase in the mitochondrial metabolism of these cells. Similarly, no statistically significant differences in apoptosis induction were detected by annexin V-FITC/PI staining, even at MNP concentrations of 150 μg Fe/ml (Fig. 2c, d). Preliminary analysis of the metabolic phenotype of MNP-treated cells using the Seahorse extracellular flux analyser technology showed enhanced mitochondrial respiration and glycolysis (Additional file 1: Fig. S1).

To analyse how the three types of MNPs interacted with Jurkat and murine T cells, we first evaluated the capacity of these cells to associate with these MNPs after co-incubation. Optimal MNP association to T cells was obtained after 2-h incubation in a reduced volume. ICP measurements showed that both cell types were able to better associate with APS-MNPs than with DMSA-MNPs or DEXT-MNPs (Fig. 2e, f). Moreover, MNP association was higher in Jurkat cells than in murine T cells. Due to its high association to T cells, APS-MNPs thus appear as the preferred candidate to study MNP-mediated retention of these cells.

**Fig. 3:**
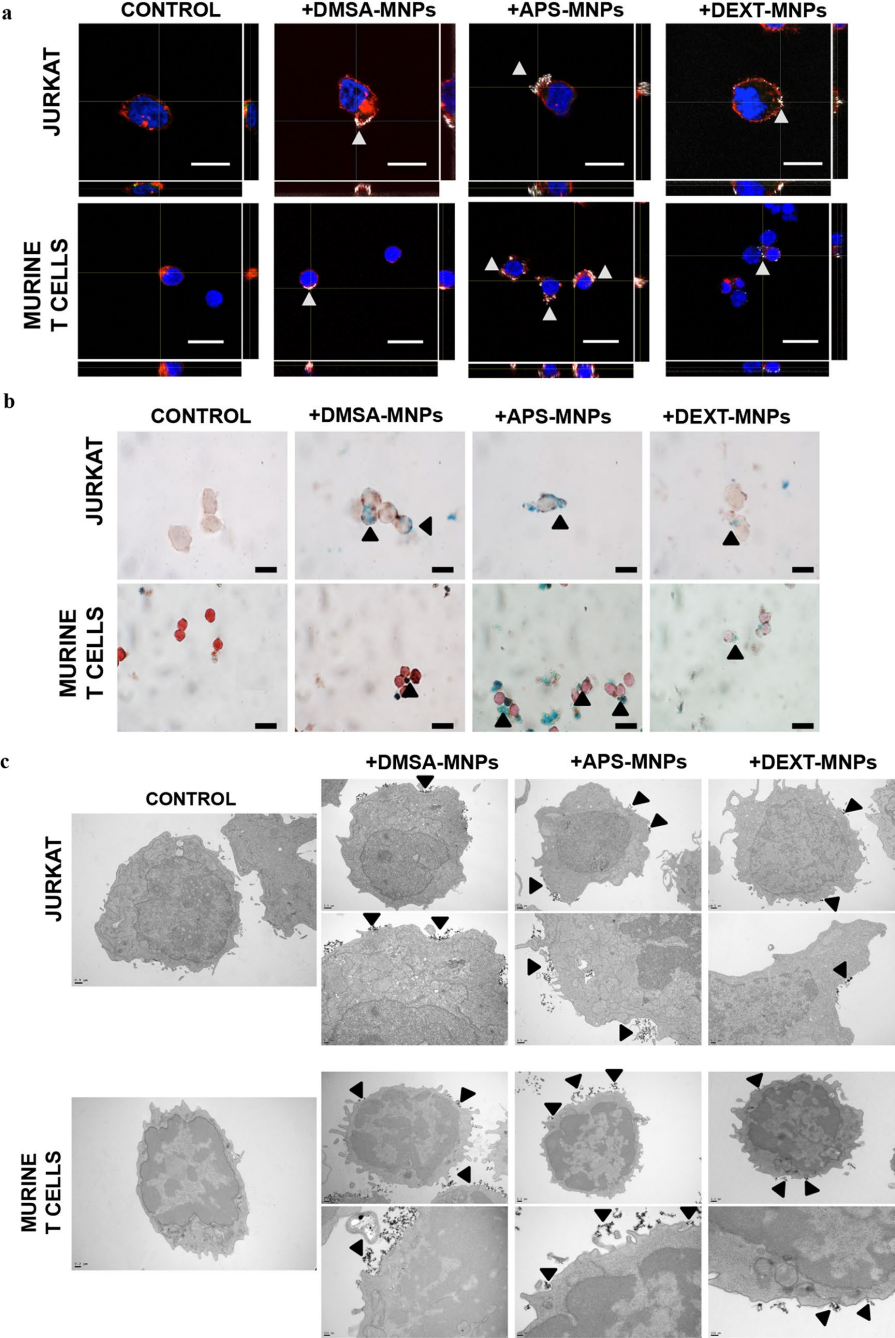
MNP subcellular localisation in Jurkat and murine primary T cells. **a** Representative images of Jurkat and murine T cells after MNP treatment acquired by confocal microscopy [cell membrane (red), MNPs (gray) and nuclei (blue)] (scale bar = 10 μm). ImageJ software was used for orthogonal projections. **b** Perls’ Prussian blue staining and neutral red counterstaining of Jurkat and murine T cells after MNP treatment (scale bar = 10 μm). **c** Representative TEM images of Jurkat and murine T cells after MNP treatment. Whole cells (top panel pictures) were imaged as well as more detailed cell parts (bottom panel pictures) to better illustrate interactions between MNPs and cell surface. Arrowheads indicate MNP aggregates attached to the cell surface

**Fig. 4:**
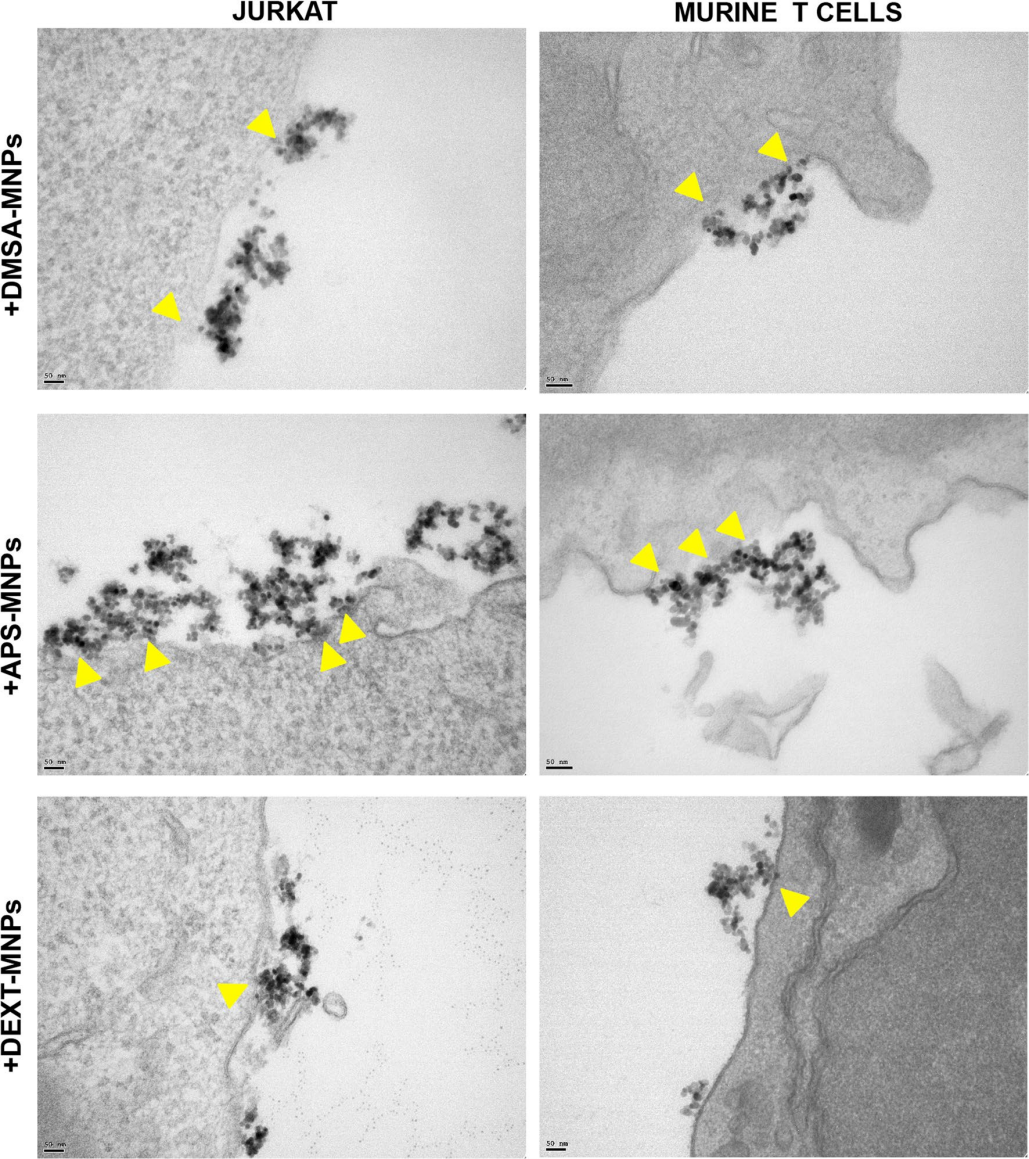
MNP association with the cell membrane of Jurkat and murine primary T cells. Representative TEM images of Jurkat and murine T cells after MNP treatment. Highly detailed images showing the interaction between MNPs and cell surface (magnification 150–×200). Arrowheads indicate MNP aggregates attached to the cell surface. Scale bar: 50 nm

We next studied MNP subcellular localization in these cells using different approaches. Pearls staining and confocal microscopy showed that MNPs, regardless of their polymeric coating, were mainly associated with the cell membrane (Fig. 3a, b). These results were confirmed by TEM (Fig. 3c). Most of the MNPs remain in the periphery of the cells, probably associated though electrostatic interactions with the membrane. TEM images at higher magnification (Fig. 4) confirmed the close interaction between the different MNPs and the cell membrane. APS-MNPs were those that seem to associate more with the surface of Jurkat and murine T cells, covering a larger cell membrane area, in line with the previous iron ICP measurements. TEM microscopy with murine T cells also revealed an increase in the number of mitochondria after MNP treatment, supporting the enhanced metabolism seen in previous assays.

### MNPs did not affect the expression of specific T cell markers but slightly impaired chemotactic response

**Fig. 5:**
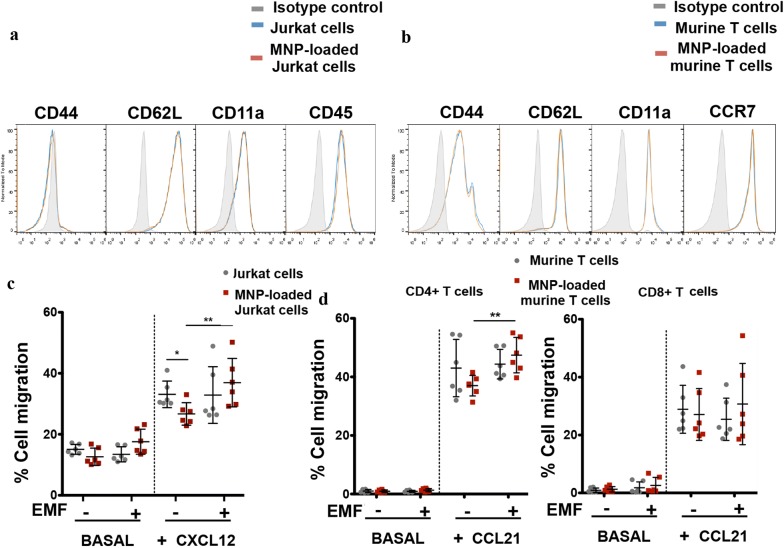
Biological effects of MNP treatment of Jurkat and murine primary T cells. Overlays of representative histograms for cell surface markers of **a** Jurkat and **b** murine T cells after MNP treatment, compared to untreated cells. Isotype/unstained control, grey fill; untreated cells, blue line; MNP-treated cells, orange line. Chemotactic response of **c** Jurkat and **d** murine T cells after treatment with MNPs. A mixture of differentially labelled MNP-free and MNP-loaded Jurkat or murine T cells (ratio 1:1) was prepared and seeded over a transwell. Their migration in the presence of chemotactic gradient of CXCL12 for Jurkat cells or CCL21 for T cells and in the presence or absence of a magnetic field was evaluated by flow cytometry after 16 h in Jurkat cells and 2 h in murine T cells. The results were normalized to input ratio. Data (mean ± SD) are representative of three-four independent experiments. Student’s t-test, *p < 0.05, **p < 0.01, ***p < 0.001

To check whether MNP association with T cells affected their effector functions, we first analysed by flow cytometry the expression levels of several important T cell surface markers. In Jurkat cells the expression of the adhesion markers CD62L and CD44, the integrin CD11a and the general leukocyte marker CD45 was evaluated (Fig. 5a). In murine T cells we analysed the expression of CD62L, CD44, CD11a and the chemokine receptor CCR7 (Fig. 5b). No differences were observed in the expression levels of these markers in Jurkat or in murine T cells when treated with APS-MNPs.

In order to effectively play their role in immune surveillance and in the body’s immune response, lymphocytes need to respond to chemotactic factors that allow them to enter and exit specific lymphatic organs and tissues [31, 32]. We therefore measured the response of MNP-loaded T cells to a chemokine gradient using transwell assay systems. MNP loading slightly impaired Jurkat cell migration in response to a CXCL12 chemokine gradient (Fig. 5c). A similar trend was also observed with primary murine T cells, although this difference was not statistically significant (Fig. 5d). This migratory defect was nonetheless corrected when a magnetic field was applied in the same direction as the biological gradient, more notably for Jurkat and CD4^+^ T cells (Fig. 5c, d). The application of an EMF probably facilitated the migration of MNP-loaded cells struggling to respond to the chemotactic gradient alone.

### Magnetic fields enhanced in vitro T cell retention in flow conditions

**Fig. 6:**
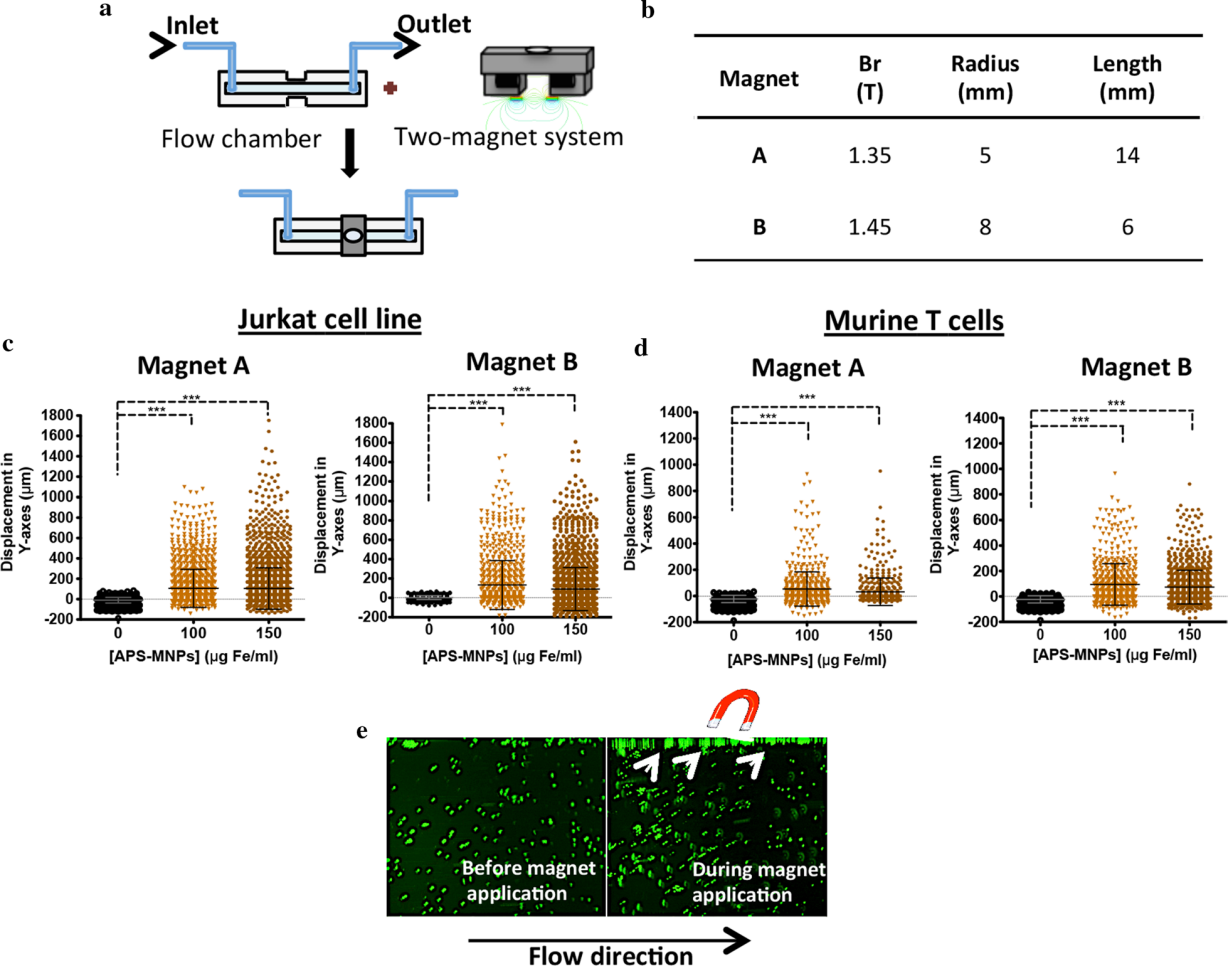
In vitro retention of MNP-loaded cells in flow chamber assays. **a** Experimental set-up for determining the magnetic in vitro retention of MNP-loaded cells. A special two-magnet system was made to apply a homogeneous magnetic gradient and ibidi flow chambers were modified to adapt them to this device. **b** Summary of the properties of the permanent neodymium magnets used. Displacement in the magnetic force direction (Y-axes) of MNP-loaded **c** Jurkat and **d** murine primary T cells for varying MNP concentrations and magnetic force conditions. Cell displacement was analysed in at least 100 cells per movie by Imaris software. Data (mean ± SD) are representative of three independent experiments. Student’s t-test, *p < 0.05, **p < 0.01, ***p < 0.001. **e** Capture of a flow chamber assay movie before and after magnetic field application. Arrows indicate the cell retention in the chamber wall near the magnet while applied

To evaluate the capacity of a magnetic field to retain MNP-loaded T cells in hemodynamic conditions we analyse whether these cells were attracted to magnets in vitro in a dynamic flow system. Flow chambers were modified so that a two magnet system that exerts a homogenous and strong magnetic gradient [33, 34] could be applied at the cell flow level (Fig. 6a). Cell retention was analysed by changing the MNP doses as well as the magnetic force used (Fig. 6b).

MNP-free cells only moved in the flow direction, as shown in the movie (Additional file 2: Movie S1), whereas cells loaded with 150 µg/ml APS-MNPs start moving towards the magnet when it was applied to the flow chamber, as shown in the movie (Additional file 3: Movie S2). T cells needed a minimal amount of MNP (> 5 pg Fe/cell in our setting) for the magnetic field produced by the 1.35 T magnets to start retaining them (Additional file 1: Fig. S2). As the amount of cell-associated MNPs increased so did the cell retention by the magnetic field (Additional file 1: Fig. S2; Fig. 6c, d). This cell accumulation was also augmented when a stronger magnet (magnet B) was applied (Fig. 6c, d). Furthermore, cells were immobilised at the chamber wall near the magnet while attached (Fig. 6e) but resume following the flow direction once the magnet was removed. Similar results were obtained with both cell types, although magnetic retention in Jurkat cells was higher than in murine T cells since they associated with more MNPs as seen in ICP measurements.

### T cell in vivo accumulation in the LNs was promoted by MNP loading and enhanced by localized EMFs application

**Fig. 7:**
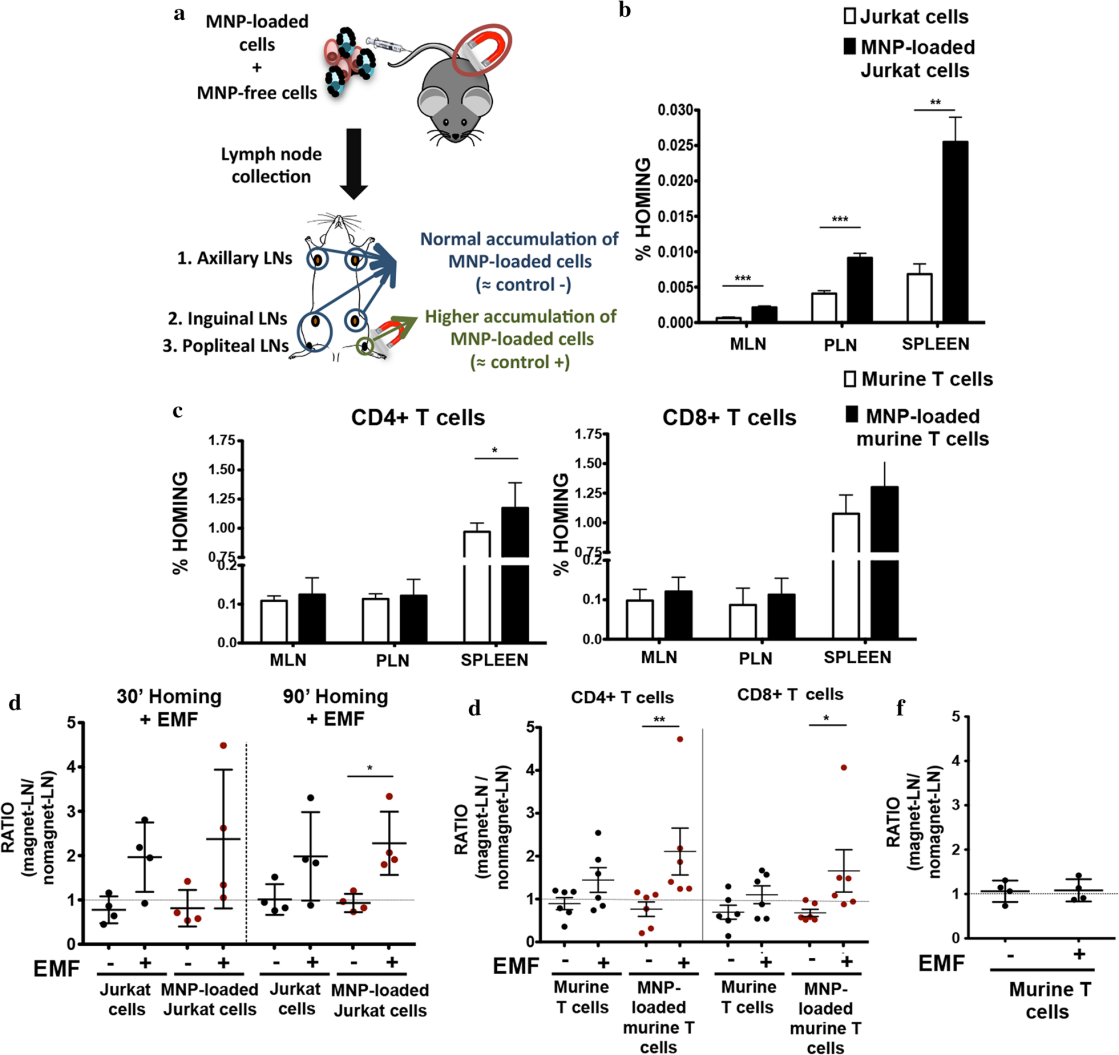
In vivo homing capacity of Jurkat and murine primary T cells after MNP treatment with and without an EMF. **a** Experimental set-up for determining the homing capacity of MNP-loaded cells compared to MNP-free cells. A mixture of differentially fluorescence-labelled MNP-free and MNP-loaded Jurkat or murine T cells (10^7^ cells; ratio 1:1) was prepared and intravenously injected into nude (Jurkat) or C57BL/6J (murine T cells) recipient mice. After 1 h, peripheral (PLN) and mesenteric (MLN) LNs and spleen were collected, processed and analysed by flow cytometry. Homing capacity of MNP-free and MNP-loaded **b** Jurkat and **c** murine T cells in the absence of an EMF, 1 h after cell injection. Data (mean ± SD) are representative of three independent experiments (n = 6). Student’s t-test, *p < 0.05, **p < 0.01, ***p < 0.001. Ratio of MNP-free and MNP-loaded **d** Jurkat and **e** murine T cells in the LN exposed to an EMF to control LN (no EMF), 20 min after intravenous injection of the cell mixture into recipient mice, normalized to the input ratio. **f** Ratio of MNP-free murine T cells, administered alone as control, in the LN exposed to an EMF to control LN (no EMF) after intravenous injection. Ratios of cell homing in magnet LN to no magnet LN (mean ± SD) are representative of three independent experiments [n = 4 (Jurkat cells), n = 6 (murine T cells)]. Student’s t-test, *p < 0.05, **p < 0.01, ***p < 0.001

Since T cells are continuously migrating into SLOs, such as LNs to maintain immune homeostasis [35], we decided to test, as a proof of concept for in vivo magnetic retention, whether the application of an EMF could affect the homing and retention of MNP loaded-T cells to a specific LN. MNP concentration (150 µg/ml APS-MNPs) and magnetic force [8 × 6 mm NdFeB permanent magnet (Br: 1.45 T)] used in in vivo studies were based on the optimal results obtained in flow chamber assays (Fig. 6c, d). A combination (ratio 1:1) of MNP-free and -loaded cells (Jurkat or murine T cells) was differentially labelled with dye Efluor 670 (red) or CFSE (green), to eliminate possible unspecific retention effects due to dye loading. Then, 10^7^ cells per mouse were intravenously inoculated in 5–6 weeks old mice (nude mice for Jurkat cells and C57BL/6 for murine T cells) and allowed to distribute in the organism. Mice were sacrificed at different timepoints post-adoptive transfer and representative lymphoid organs collected (axillary, inguinal, popliteal, mesenteric LNs and spleen depending on the experiments). In some experiments, an EMF was applied over a popliteal LN to promote specific lymphoid cell accumulation (Fig. 6a). In vivo homing in the absence of an EMF was first tested to determine whether MNP loading impaired T cell trafficking towards SLOs. Irrespective of their origin (either the human T cell line Jurkat or primary murine T cells) MNP-loaded T cells were found in a higher number than MNP-free T cells in the collected peripheral and mesenteric LNs and in the spleen (Fig. 7b, c). This difference was more remarkable in the case of Jurkat cells (Fig. 7b), and at later timepoints (data not shown).

**Fig. 8:**
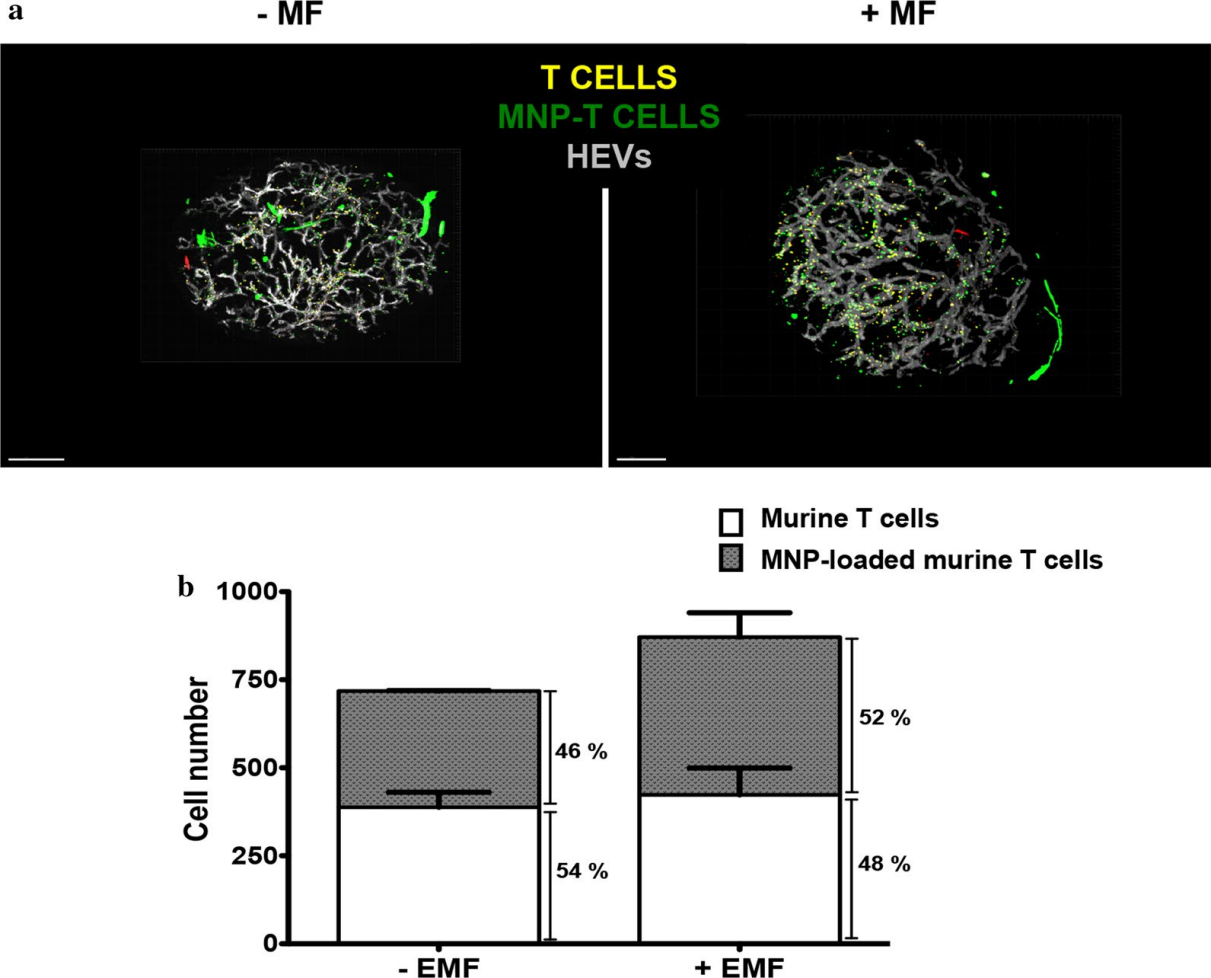
Distribution of MNP-free and MNP-loaded murine primary T cells in the popliteal LN in the absence or the presence of an EMF. **a** SPIM images of the popliteal LNs, 20 min after intravenous injection of a mixture of MNP-free (yellow) and MNP-loaded (green) murine T cells (ratio 1:1) into C57BL/6J recipient mice, exposed (right panel) or not (left panel) to an EMF. High endothelial venules (HEVs) are labelled in grey. **b** Quantification of the total transferred MNP-free and MNP-loaded murine T cells in SPIM images and the percentage of each cell type found in the absence or the presence of an EMF. Data are representative of 2 independent experiments

After verifying that MNP treatment did not impair T cell homing capacity, these experiments were repeated with an EMF applied to a popliteal LN to potentially enhance cell retention at this location. Flow cytometry analyses after LN collection and disaggregation revealed that similar amounts of cells were found in pairs of matching lymph when no EMF was applied. EMF application increased the amount of cells found on the popliteal LN where the magnet was applied (Fig. 7d, e). The slight increase also detected in MNP-free cells in the LN with EMF application was probably due to their interaction with MNP-treated cells in the cell mixture prior to injection, resulting in MNP transfer and/or aggregation of treated and untreated cells. Indeed, control inoculations of MNP-free cells alone showed no differential increase of this cell population in the LN over which the EMF was applied (Fig. 7f). Popliteal LNs were also imaged using SPIM. Image analysis revealed that larger numbers of transferred cells loaded with MNPs were retained in the LN exposed to a magnet than in non-exposed one (Fig. 8). These results confirm the results obtained in our in vivo homing studies.

### MNPs and EMFs reduced T cell speed in popliteal LNs

**Fig. 9:**
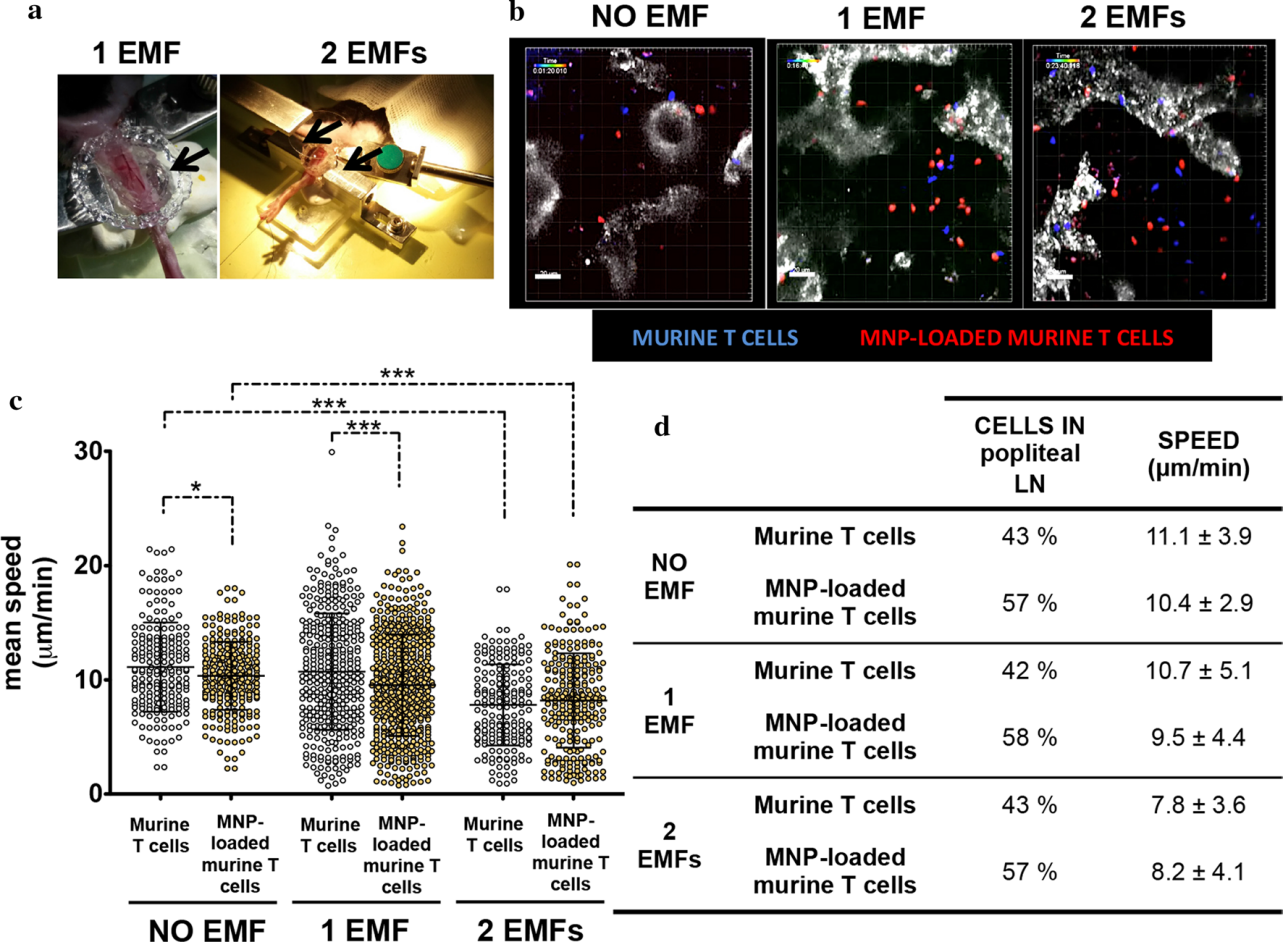
In vivo behaviour of MNP-free and MNP-loaded murine primary T cells in the popliteal LN in the absence or the presence of an EMF. **a** Experimental set-up of the 2PM assays, showing the exposure of the popliteal LN to the different EMF conditions. **b** Capture of the 2PM movies after intravenous injection of a mixture of 10^7^ cells, differentially labelled MNP-free (blue) and MNP-loaded (red) murine T cells into C57BL/6J recipient mice, in the absence of an EMF and in the presence of one or two magnets. HEVs are labelled in grey. **c** Quantification of cell speed in all conditions with Imaris software. Data (mean ± SD) are representative of two or three independent experiments. Student’s t-test, *p < 0.05, **p < 0.01, ***p < 0.001. **d** Summary of the analyses of 2PM movies. Ratio (%) of unloaded to MNP-loaded cells and cell speed (mean ± SD) in the popliteal LN are representative of two or three independent experiments

Once magnetic retention of MNP-loaded T cells in the LNs was confirmed, we also wanted to study T cell behaviour in vivo and in real time. For this, intravital two-photon microscopy of popliteal LNs was performed in the absence of EMF or in EMF presence on one side or on both sides of the popliteal LN to increase magnetic field strength (Fig. 9a, b). A combination of MNP-loaded and MNP-free cells (ratio 1:1) was inoculated just prior to recording and cell speed while traveling through the popliteal LN was analysed with the Imaris software. Image analysis from the recorded movies (Additional file 4: Movie S3; Additional file 5: Movie S4; Additional file 6: Movie S5) showed that MNP-loaded cells had a 10% reduction in their speed compared to MNP-free cells even in the absence of EMF (Fig. 9c, d). No preferential location or directionality was observed in these experiments, even in the presence of an EMF (Additional file 1: Fig. S3). Image analysis nonetheless confirmed that the MNP-loaded T cells were better retained in the popliteal LN than MNP-free cells (Fig. 9d). EMF application further reduced MNP-loaded T cell speed in the popliteal LN, and this was exacerbated by the application of 2 EMF. Surprisingly the speed of MNP-free cells was also reduced in the popliteal LN when an EMF was applied (Fig. 9c, d). These could nonetheless be artefactual effects comparable to those detected in in vivo homing experiments (Fig. 7d–f) due to MNP transfer from MNP-loaded to MNP-free cells during inoculum preparation. It thus appears that MNP loading of T cells can promote cell retention in LNs and that circulation speed through the lymphoid organ can be reduced by EMF application.

## Discussion

To evaluate the possibility of using MNPs to magnetically direct or retain T cells to a region of interest, we synthesized and characterized MNPs with coatings that provide different surface charges, and we have studied their effects on T cell. The MNPs used in this study were produced by the coprecipitation method and had an average core diameter of 12.5 nm. FTIR spectra and DLS measurements confirmed coating procedure succeeded in creating monodispersed MNP preparations with different surface charges. MNP treatment did not cause any toxicity in the human T cell line Jurkat or in murine primary T cells in the conditions tested. MNP-treated murine primary T cells, however, presented a higher mitochondrial respiration and glycolysis. This could be associated with an activated state in T cells, since aerobic glycolysis increases during the transition to effector T cells. Moreover, glycolytic catabolism is also associated with T cell functionalities such as T_reg_ and memory T cells [36].

Microscopy analysis showed that unlike macrophages, mesenchymal or malignant cells, which are able to internalize different type of MNPs [11, 37–39], including the ones used in the present study (Additional file 1: Fig. S4) [40], T cells are apparently unable to do so. MNPs remain on the cell surface in close contact with cell membrane. T cells have typically low phagocytic capacity [41] with large nuclei and reduced cytoplasm, which could impair MNP entry inside the cytoplasm. Moreover, it has been also described that transfection of such cell types has always presented many difficulties and limitations, being a really inefficient process [42–46]. The low endocytic capacity of lymphoid cells has been already reported [47, 48] and a special nanoparticle design is usually required to improve internalization by these nonphagocytic cells [49]. For example, some studies indicate that the uptake of NPs can be increased by coupling the NP to peptides or proteins, such as Tat protein of HIV, that exhibit a high permeability [50–52]. Microscopy analysis and cell-associated iron quantification showed that APS-MNPs were the MNPs most associated with T cells and they were therefore selected to perform subsequent magnetic retention experiments. DMSA-MNPs and DEXT-MNPs have negative and neutral charges respectively, whereas APS-MNPs are positively charged which probably favour their interaction with the negative potential of the cell membrane. There is a general tendency to assume that positively charged nanoparticles interact further with cell membranes, possibly due to electrostatic interactions [53–60]. In this study, the positive MNPs (APS-MNPs) were the ones that interacted most with the membrane of the lymphoid cells, which correlates with what is described in the literature. Besides, some of the neutral and negative MNPs (DEXT- and DMSA-MNPs respectively) also interact but with smaller areas of the cell surface. This could be explained by the fact that, although cell membrane is, in general, negatively charged, it also presents areas with cationic sites allowing the binding of negatively charged NPs resulting in the clustering of the NPs around those domains [61].

Cytometric analyses of MNP-treated T cells showed that APS-MNPs caused no differences in the expression levels of important cell surface markers such as CD62L, CD44, CD11a or CCR7 in Jurkat and primary T cell. These data are in consonance with previous reports showing that MNPs have no significant effect on the expression levels of surface markers of a variety of cells [62–66]. MNPs can trigger cellular activation in some cases [39, 67, 68] but this is mostly due to the nanoparticle design [69].

Chemotaxis is critical to T cell biology as it guides T cell movement across tissues and to the regions where their activity is required. MNP loading slightly decreased chemotactic responses. Our data nonetheless indicate that the adequate application of an EMF could correct this defect, probably because the magnetic gradient facilitated the migration of these MNP-loaded cells. Similar results are reported in dendritic cells [12, 66], which indicate that MNP loading of immune cells could affect their chemotaxis. One of the main concerns of the present study was to evaluate the capacity of a magnetic field to promote the retention of MNP-loaded T cells in the presence of flow forces similar to the ones found in blood vessels. Our results showed that a minimal amount of MNPs was required for T cells to be retained by an EMF. This magnetic retention increases with the amount of MNPs associated to the cells as well as with the magnetic field magnitude. Other studies also reported that these two parameters are critical for this magnetic targeting to occur [70, 71].

The in vivo analysis of T cell homing capacity in the presence or absence of MNPs revealed a higher amount of MNP-loaded T cells retained in the LNs, indicating that the presence of MNPs on the surface of T cells could increase by itself their retention in SLOs. MNP-loaded cells, however, did not show any preferential location or distribution for a specific SLO. The preferential accumulation of MNP-loaded T cells in lymphoid tissues even in the absence of a magnetic field has the potential to favour T cell interactions with accessory cells in these organs where T cell activation occurs. This property of MNP-loaded T cells could be used to promote T cell responses to specific antigens as well as to modulate the immune response in a pathological context. For example, for therapies based on adoptive transfer of regulatory T cells (T regs), approaches enhancing the LN trafficking and retention of these T regs could be beneficial to prevent immune-mediated diseases [72]. The strategic LN enrichment of antigen-specific T regs is crucial in controlling autoreactive effector T cells (T effs) and autoimmunity [73, 74]. For instance, it has been described that the accumulation of regulatory T cells in a LN near an ovary, counteracts the pathogenic immune response that takes place during ovarian autoimmune disease, inhibiting therefore its development [74]. Besides, there are studies showing that the recirculation of T regs in mesenteric LNs contributes to the downregulation of intestinal inflammation [75], that their accumulation in the draining LN of the lung correlates with the resolution of chronic asthma in murine models [76] and also that the migration of T regs to specific draining LNs is required to suppress the alloimmune response [77] and is capable to induce suppression of arthritis [78]. Therefore, the modulation of T cell trafficking could have interest in the treatment of autoimmune diseases. This preferential accumulation of MNP-loaded T cells in lymphoid tissues was also confirmed in the SPIM analyses of the LNs. There are some reports that analyse the biodistribution of MNP-labelled cells in different organs at short time points by magnetic resonance imaging [79–81]. Dodd et al. showed that most of naïve and memory T cells migrated to the spleen and LNs but also to the lungs and liver after adoptive transfer [81]. It is also described that in a tumour model, MNP-labelled CD8^+^ T cells accumulated preferentially in the spleen and lymphoid tissues as well as in the tumour but also in the liver and in the lung [79, 80]. In this in vivo analysis, the application of an EMF favoured the retention of MNP-loaded transferred cells in the LN exposed to the magnet, indicating that, after reaching the LN, more MNP-loaded T cells were retained due to the presence of the EMF. Similar results have been obtained using other different cell types and regions of interest [12, 18, 38, 65, 66, 71, 82]. Our results reinforce the idea of magnetic targeting as a way to improve specific cell accumulation and extend its application to T lymphocytes, a relatively unexplored field.

Two-photon microscopy intravital in vivo behaviour of MNP-loaded T cells in the popliteal LNs confirmed the increased retention of MNP-loaded T cells in lymphoid organs and revealed a 10% reduction in their speed compared to MNP-free T cells. The increased retention of MNP-loaded T cells in lymphoid organs even in the absence of EMF (Fig. 7b, c) could thus be due to a reduced circulating speed that would prolong cell interaction with lymphoid tissue vasculature and facilitate retention. Unexpectedly, the speed of both MNP-loaded and –free T cells was reduced when applying an EMF. This could be partly due to the interaction between loaded and unloaded cells in the mixture prior to inoculation as previously demonstrated (Fig. 7d–f), resulting in the transfer of some MNPs to the unloaded T cells. Additionally, it has been previously described that cells associated with MNPs present a greater tendency to aggregate [83], so MNP-loaded cells could probably interact further with other surrounding cells, treated or not with MNPs. Alternatively, moderate static magnetic fields can influence biological systems, mostly due to alterations of the transmembrane ion flux and the membrane phospholipids [84–86]. Preliminary calcium imaging assays did not show significant alterations in calcium fluxes, essential for T cell functionality, neither in the maximum response nor the time to achieve it, in the presence of MNP in the case of Jurkat cells (Additional file 1: Fig. S5a, c). Murine T cells, however, showed a decrease in both, the maximum response and the mean response after MNP loading (Additional file 1: Fig. S5b, d). Further studies would be needed to confirm this finding and determine its biological significance. Biological effects supposedly depend more on the magnitude of the magnetic field gradient than on its strength [87]. This could explain the increased reduction in cell speed obtained in intravital experiments when two magnets were placed over the popliteal LN. This reduced speed could also have some interest. It has been reported that the duration of the interaction between dendritic cells and T cells in the LNs affects the specificity and potency of the T cell responses [88–91]. The decrease in the speed of T cells in the LN could serve to favour their interaction with the dendritic cells and the generation of more powerful T cell responses with a greater affinity. Cell path analysis in these experiments indicated that MNP loading does not influence the lymphocyte trajectory inside de LN when an EMF is applied, although further analysis over longer time periods and with increased EMF strength could be necessary to detect trajectory changes due to the complex tissue architecture of LN. Some of these effects could also be masked by MNP transfer to unloaded cells during inoculum preparation.

## Conclusions

T lymphocyte migration is a strongly regulated process, essential in the development of potent and effective immune responses against different antigens. In the present study, we successfully show that the use of MNPs and EMFs can guide and retain T lymphocytes to a target region of interest without critically affecting crucial biological aspects of these cells. We demonstrate that MNP-loaded murine T cells can be magnetically retained in the LNs, which could be useful to modulate immune response in a pathological context. This strategy could potentially be applied to other regions of interest, such as a tumour, to enhance the antitumoral immune response led by activated T cells.

Thus, our study describes an interesting and affordable approach to promote T cell retention that could be implemented in adoptive cell transfer therapies. This magnetic manipulation of T cell localisation could help improve their efficacy in autoimmunity and cancer treatment.

## Additional files

**Additional file 1: Fig. S1.** Metabolic phenotype of murine primary T cells after MNP treatment. **Fig. S2.** MNP dose-dependent in vitro retention of MNP-loaded Jurkat cells. **Fig. S3.** MNP-free and -loaded T cells’ trajectories inside de LN in the absence or the presence of an EMF. **Fig. S4.** MNP subcellular localisation in the murine macrophage RAW264.7 cell line. **Fig. S5.** Calcium fluxes after MNP treatment.

**Additional file 2: Movie S1.** Image sequence showing MNP-free T cells (green) in flow chamber assays before and after the application of an EMF in the upper part of the chamber.

**Additional file 3: Movie S2.** Image sequence showing MNP-loaded T cells (green) in flow chamber assays before and after the application of an EMF in the upper part of the chamber.

**Additional file 4: Movie S3.** 2PM image sequence showing MNP-free murine T cells (blue) and MNP-loaded murine T cells (red) at early time points after t cell transfer in the popliteal LN, in the absence of an EMF. Bar, 20 µm.

**Additional file 5: Movie S4.** 2PM image sequence showing MNP-free murine T cells (blue) and MNP-loaded murine T cells (red) at early time points after t cell transfer in the popliteal LN, in the absence of a single EMF. Bar, 20 µm.

**Additional file 6: Movie S5.** 2PM image sequence showing MNP-free murine T cells (blue) and MNP-loaded murine T cells (red) at early time points after t cell transfer in the popliteal LN, in the presence of a double EMF. Bar, 20 µm.
