# Supplementary material for: T cells loaded with magnetic nanoparticles are retained in peripheral lymph nodes by the application of a magnetic field
Source: J Nanobiotechnology. 2019 Jan 22;17:14. doi: 10.1186/s12951-019-0440-z (PMC6341614; doi:10.1186/s12951-019-0440-z)
Supplement: Supplementary file 1 — Additional file 1: Fig. S1. Metabolic phenotype of murine primary T cells after MNP treatment. Fig. S2. MNP dose-dependent in vitro retention of MNP-loaded Jurkat cells. Fig. S3. MNP-free and -loaded T cells’ trajectories inside de LN in the absence or the presence of an EMF. Fig. S4. MNP subcellular localisation in the murine macrophage RAW264.7 cell line. Fig. S5. Calcium fluxes after MNP treatment. [file 12951_2019_440_MOESM1_ESM.docx]

**ADDITIONAL FILE 1**

**T cells loaded with magnetic nanoparticles are retained in peripheral lymph nodes by the application of a magnetic field**

**Laura Sanz-Ortega^a^, José M. Rojas^a,c^, Ana Marcos^b,d^, Yadileiny Portilla^a^, Jens V. Stein^b,d^, Domingo F. Barber^a,^[[1]](#footnote-1)^*^**

^a^Department of Immunology and Oncology, and NanoBiomedicine Initiative, Centro Nacional de Biotecnología (CNB)-CSIC, Darwin 3, Cantoblanco, 28049 Madrid, Spain

^b^Theodor Kocher Institute, University of Bern, 3012 Bern, Switzerland

^c^Current address: Animal Health Research Centre (CISA)-INIA, Instituto Nacional de Investigación y Tecnología Agraria y Alimentaria, Valdeolmos, 28130 Madrid, Spain

^d^Current address: Department of Oncology, Microbiology and Immunology, Section of Medicine, University of Fribourg, 1700 Fribourg, Switzlerland

*Address correspondence to [dfbarber@cnb.csic.es](mailto:dfbarber@cnb.csic.es)

**FIGURES**

**
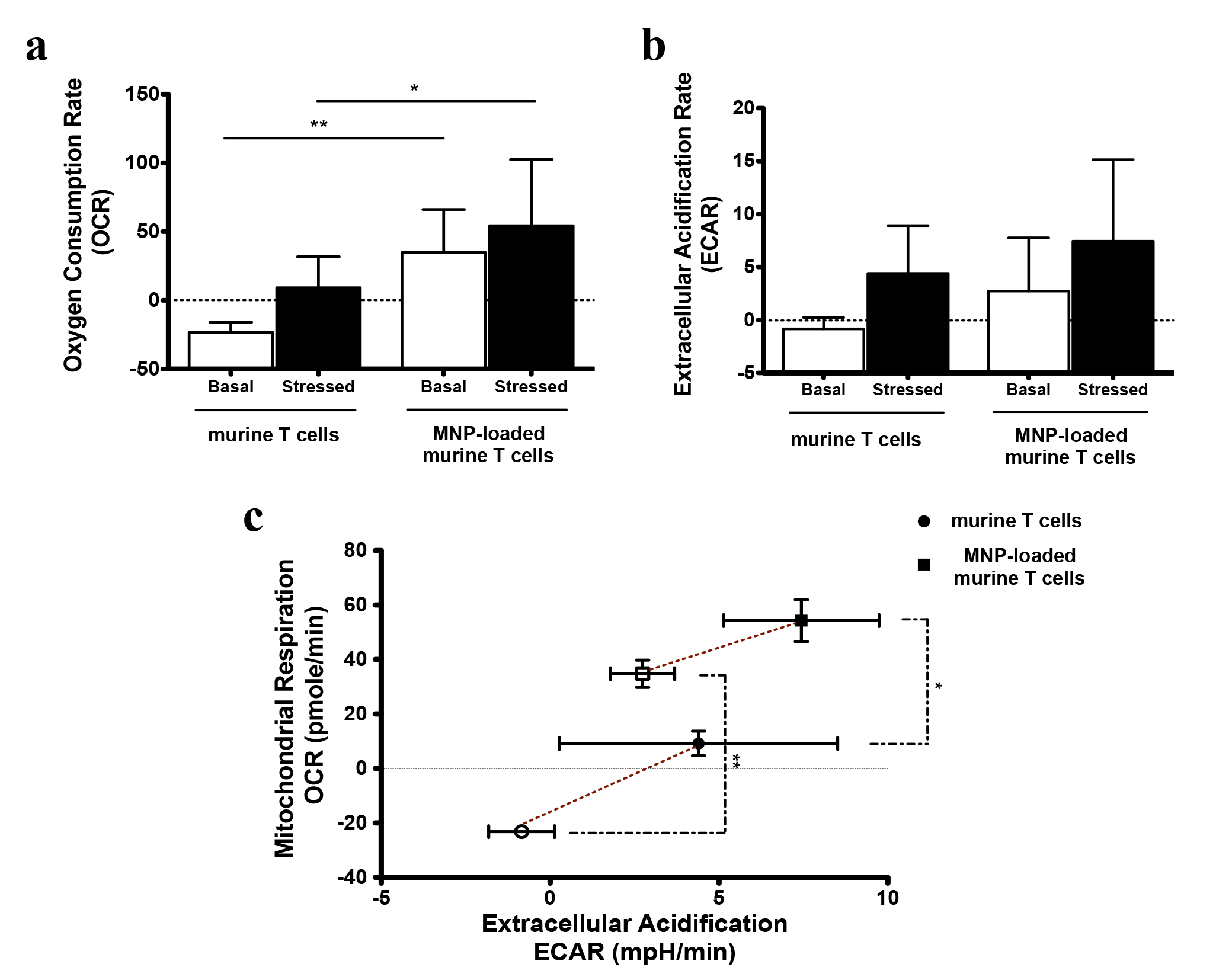
**

**Fig.S1** Metabolic phenotype of murine primary T cells after MNP treatment. Murine T cells were treated or not with MNPs and analysed using the Seahorse XFp Analyzer. a) Oxygen consumption rate (OCR) and b) Extracellular acidification rate (ECAR) measurements in MNP-free and -loaded murine T cells. c) Cell energy potential (OCR vs ECAR) in MNP-free and loaded murine T cells. Data (mean ± SD) are representative of three independent experiments. Student’s t-test, * p < 0.05, ** p < 0.01, *** p < 0.001


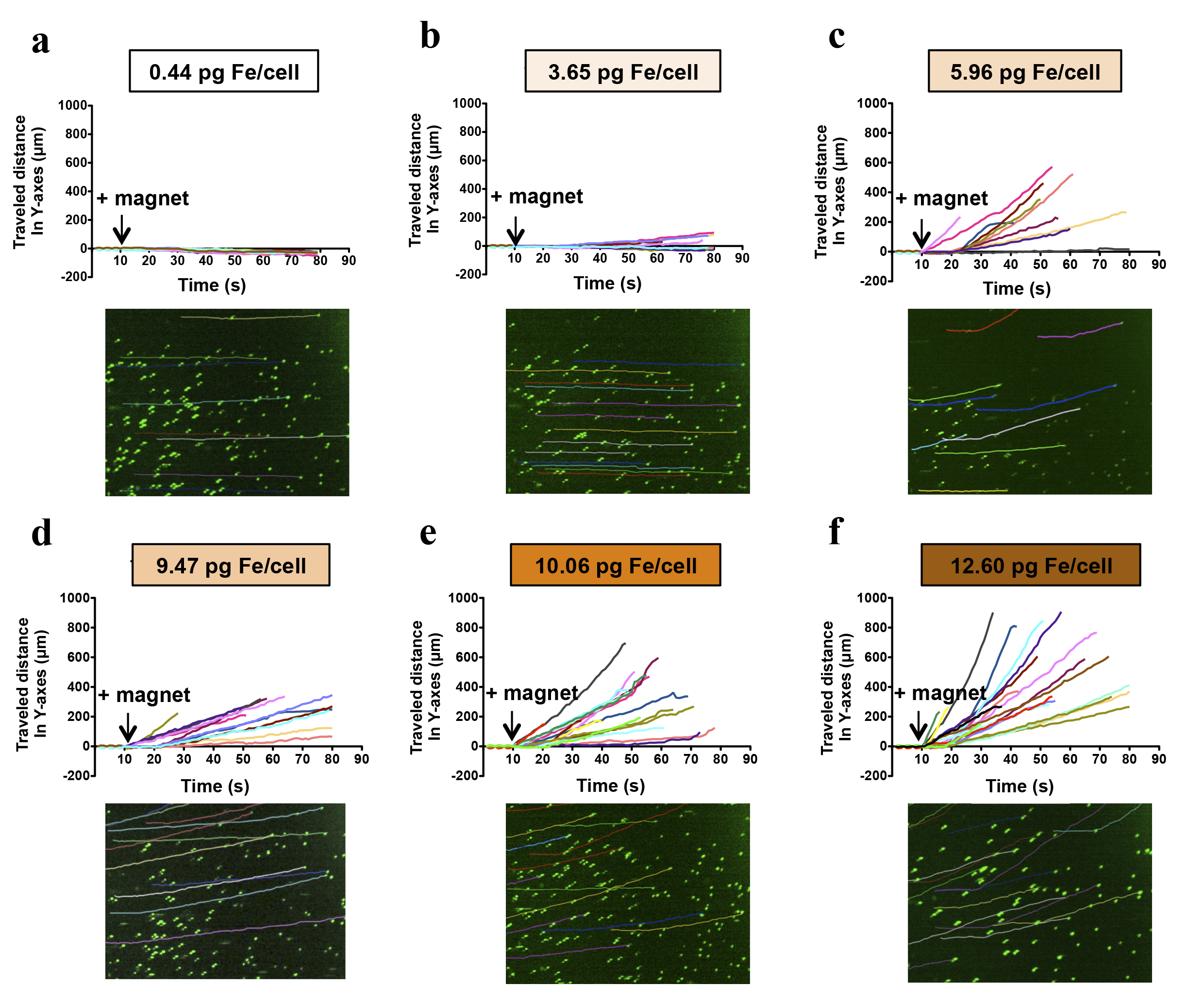


**Fig.S2** MNP dose-dependent *in vitro* retention of MNP-loaded Jurkat cells. Jurkat cells were treated with increasing MNP concentrations and their magnetic *in vitro* retention was evaluated in flow chamber assays by applying magnetic force with 1.35 T magnets. Displacement quantifications in the magnetic force direction (Y-axes) as well as a capture of each movie are shown in the different panels (a-f). Iron cell association was measured in each cell preparation as indicated in the top part of every condition

**
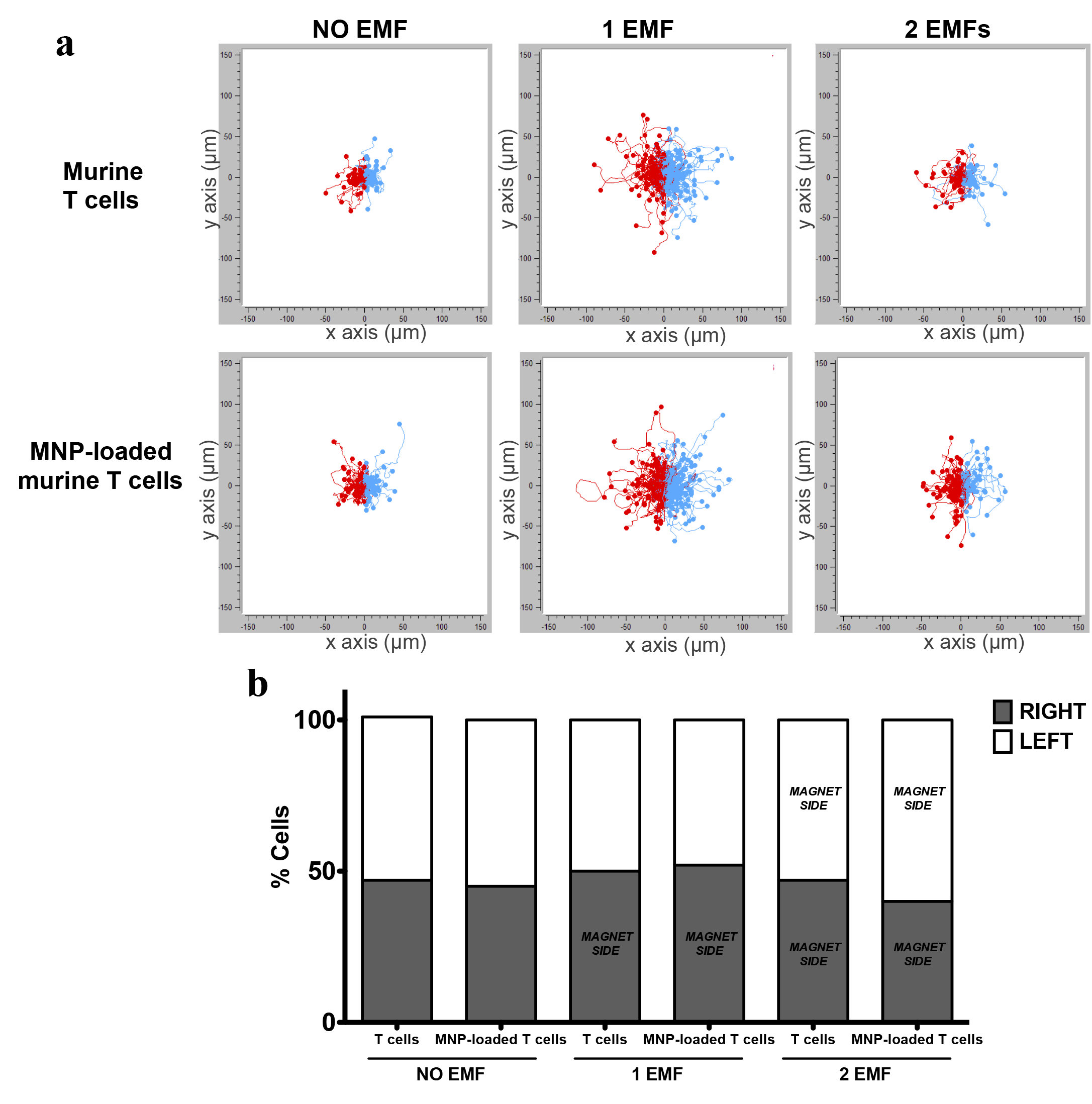
**

**Fig.S3** MNP-free and –loaded T cells’ trajectories inside de lymph node in the absence or the presence of an EMF. a) MNP-free and –loaded murine T cells’ paths inside the LN during the 2PM assays (red: right direction, blue: left direction), analysed with the Chemotaxis and Migration tool (Ibidi). b) Percentage of cells migrating to each side, in the presence or absence of an EMF

**
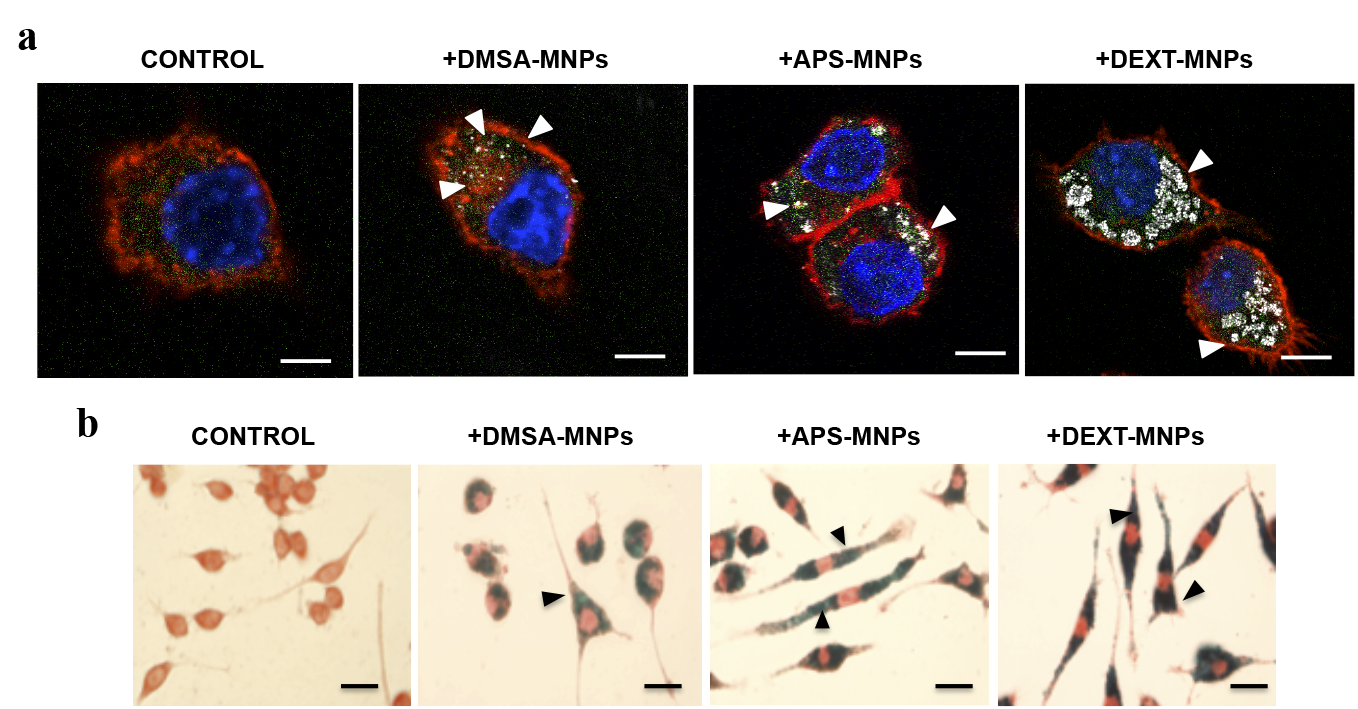
**

**Fig.S4** MNP subcellular localisation in the murine macrophage RAW264.7 cell line. Representative images of RAW264.7 cells after MNP treatment by a) confocal miroscopy (cell membrane (red), MNPs (gray), lysosomes (green) and nuclei (blue)) (scale bar = 25 μm) and b) Perls’ Prussian blue staining and neutral red counterstaining (scale bar = 20 μm). Arrowheads indicate MNPs inside the cells and mainly associated to the lysosomes

**Fig.S5** Calcium fluxes after MNP treatment. Kinetics of calcium flux in MNP-free and –loaded a) Jurkat and b) murine primary T cells. Representative images of MNP-free and -loaded a) Jurkat and b) murine T cells during the assay before and after adding ionomycin. Fluorescence intensity is indicated by a linear scale (256 shades) shown at right. Summary of the main parameters after image analysis in MNP-free and –loaded c) Jurkat and d) murine T cells. Data (mean ± SD) are representative of three independent experiments

1. [↑](#footnote-ref-1)
